# Interleukin-18 in Inflammatory Kidney Disease

**DOI:** 10.3389/fmed.2021.639103

**Published:** 2021-03-01

**Authors:** Yasuaki Hirooka, Yuji Nozaki

**Affiliations:** ^1^Department of Rheumatology, Kindai University Nara Hospital, Nara, Japan; ^2^Department of Hematology and Rheumatology, Kindai University School of Medicine, Osaka, Japan

**Keywords:** IL-18, inflammatory kidney disease, inflammation, IL-1, COVID-19

## Abstract

Interleukin (IL)-18, a member of the IL-1 superfamily, is a pro-inflammatory cytokine that is structurally similar to IL-1β. IL-18 promotes the production of interferon gamma (IFN-γ) and strongly induces a Th1 response. IL-18 drives the same myeloid differentiation factor 88 (MyD88)/nuclear factor kappa B (NF-κB) signaling pathway as IL-1β. In physiological conditions, IL-18 is regulated by the endogenous inhibitor IL-18 binding protein (IL-18BP), and the activity of IL-18 is balanced. It is reported that in several inflammatory diseases, the IL-18 activity is unbalanced, and IL-18 neutralization by IL-18BP is insufficient. IL-18 acts synergistically with IL-12 to induce the production of IFN-γ as a Th1 cytokine, and IL-18 acts alone to induce the production of Th2 cytokines such as IL-4 and IL-13. In addition, IL-18 alone enhances natural killer (NK) cell activity and FAS ligand expression. The biological and pathological roles of IL-18 have been studied in many diseases. Here we review the knowledge regarding IL-18 signaling and the role of IL-18 in inflammatory kidney diseases. Findings on renal injury in coronavirus disease 2019 (COVID-19) and its association with IL-18 will also be presented.

## Introduction

Inflammation is a defense mechanism that is caused by harmful stimuli and conditions such as infection and tissue injury (1). Innate immunity is the host's first line of defense against pathogens and is activated by pattern recognition receptors (PRRs). PRRs recognize pathogen-associated molecular patterns (PAMPs) and danger-associated molecular patterns (DAMPs) that are common to pathogens. There are several classes of PRRs, including Toll-like receptors (TLRs), C-type lectin receptors, nucleotide-binding oligomerization domain-like receptors (NLRs), retinoic acid-inducible gene-I-like receptors (RLRs), and absent in melanoma 2 (AIM2)-like receptors. The inflammasome, a multiprotein complex formed intracellularly in response to PAMPs and DAMPs, converts procaspase-1 to active caspase-1 and induces pro-inflammatory cytokines such as interleukin 1beta (IL-1β) and IL-18 (2).

The NLR family member leucine rich repeat and pyrin domain containing 3 (NLRP3) forms the NLRP3 inflammasome together with the adapter molecules apoptosis-associated speck-like protein containing a caspase recruitment domain (ASC) and procaspase-1 and activates caspase-1 to processes IL-1β and IL-18 to the bioactive mature form (3, 4). The NLRP3 inflammasome has been implicated in the pathogenesis of many diseases, including microbial pathogens, inflammatory diseases, cancer, and metabolic and autoimmune disorders (3, 4), and it has also been implicated in various kidney diseases (5). A member of the IL-1 superfamily, IL-18 is a pro-inflammatory cytokine that is structurally similar to IL-1β (6, 7). IL-18 promotes the production of interferon gamma (IFN-γ) and strongly induces a Th1 response (8).

In recent years, the biological and pathological roles of IL-18 have been studied in many diseases. Inflammation underlies the pathogenesis of many acute or chronic kidney diseases, and IL-18 plays an important role. This paper focuses on the roles of IL-18 in inflammatory kidney diseases. We review the current knowledge regarding IL-18 signaling, and we outline the existing evidence about the roles of IL-18 in inflammatory kidney diseases. We are in the midst of an epidemic of coronavirus disease 2019 (COVID-19). Findings on renal injury in cases of COVID-19 and its association with IL-18 will also be presented.

## The Production and Processing of IL-18

IL-18 was initially identified as IFN-γ-inducing factor (IGIF) in sera from *Propionibacterium acnes*-primed and lipopolysaccharide (LPS)-challenged mice (9–11). Although IL-18 and IL-1β share only about 17% sequence homology, they have a common β-pleated sheet structure (12, 13). IL-18 is produced by macrophages, dendritic cells, epithelial cells, keratinocytes, chondrocytes, osteoblasts, synovial fibroblasts, and adrenal cortex cells, and it plays an important role in inflammatory pathology (7, 13, 14). In the kidney, the predominant source of IL-18 production is tubular epithelial cells (15–17). IL-18 gene expression may be enhanced by stimulation with microbe products such as LPS and by cytokines such as IFN-α/β/γ and TNF-α (13, 18, 19). IL-18 is stored intracellularly as a biologically inactive 24-kDa precursor (pro-IL-18), similar to IL-1β, and is secreted extracellularly as the 18-kDa bioactive mature molecule after being cleaved by caspase-1. Nitric oxide suppresses the secretion of IL-1β and IL-18 by inhibiting caspase-1 (20). An inhibitor of mammalian target of rapamycin (mTOR), rapamycin is widely used as an autophagy inducer (21). The induction of autophagy by rapamycin can suppress the production and secretion of IL-1β and IL-18 and limit excessive inflammation (21).

## IL-18 Receptor and Signal Transduction

**Figure 1:**
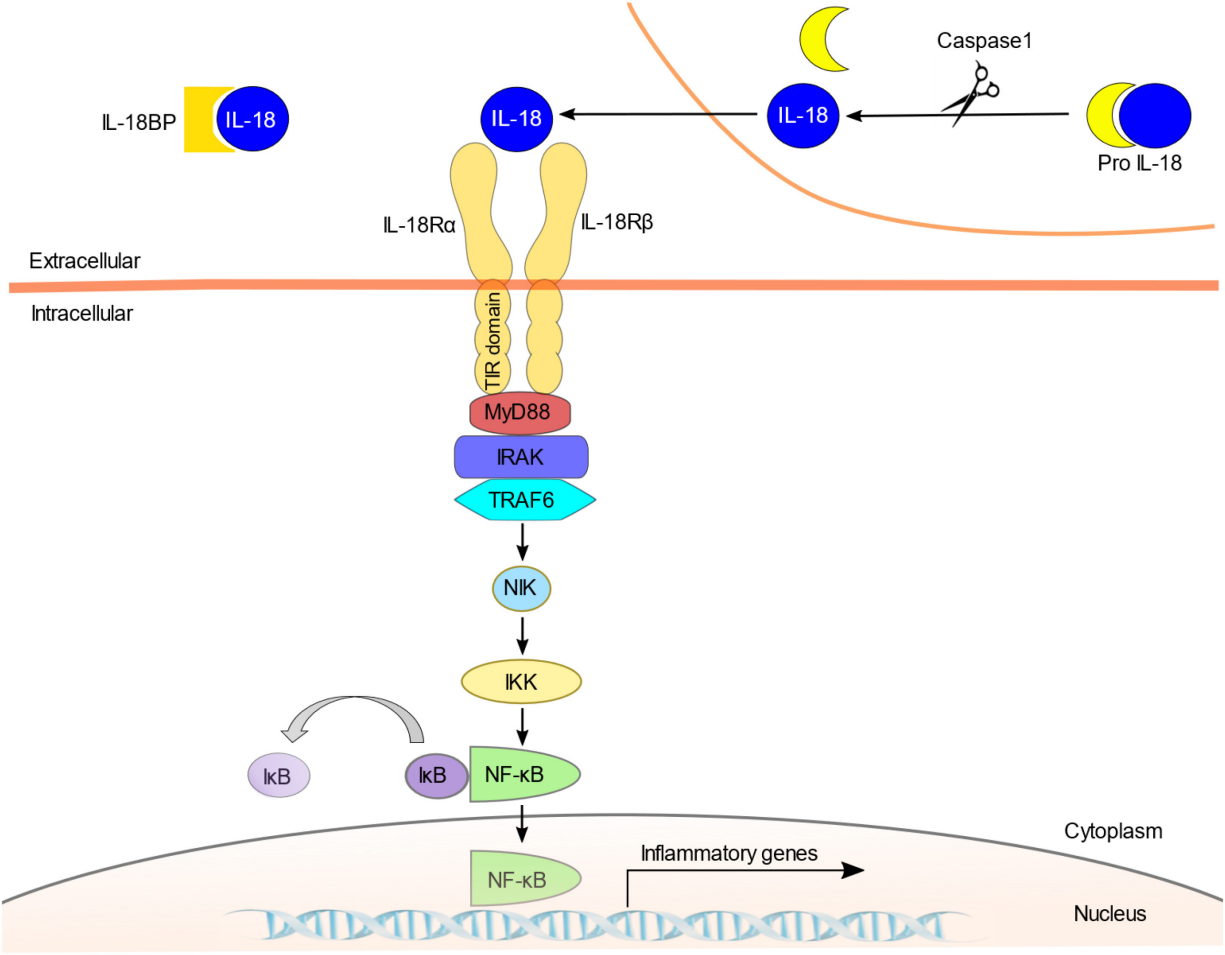
The IL-18 signaling pathway. IL-18 is stored intracellularly as biologically inactive pro-IL-18 and is secreted extracellularly as the bioactive mature molecule after being cleaved by caspase-1. IL-18 is regulated by the endogenous inhibitor IL-18BP. Since IL-18BP has high affinity for IL-18, IL-18BP binds preferentially to IL-18 and inhibits binding to the IL-18R. IL-18 first binds to the IL-18Rα; however, this binding is low-affinity, and the IL-18Rβ chain is recruited to form a high-affinity heterodimeric complex. The activation of IL-18R recruits MyD88 to the TIR domain and anchors IRAK. Phosphorylated IRAK activates TRAF6, and which in turn phosphorylates NIK. This is followed by the activation of IKK and finally NF-κB, which initiates the transcription of target genes such as IFN-γ.

The IL-18 signaling pathway is illustrated in **Figure 1**. IL-18 recognizes a heterodimeric receptor that consists of IL-18 receptor (R) α- and β-chains (22). IL-18Rα, also known as IL-1 receptor-related protein (IL-1Rrp), binds specifically to the extracellular IL-18 at the cell surface. However, its affinity is low. IL-18Rβ (i.e., accessory protein-like [AcPL]) is recruited to form a high affinity binding and activate intracellular signaling pathway (23, 24). IL-18R is expressed in most types of cells, including T cells, natural killer (NK) cells, macrophages, dendritic cells, neutrophils, basophils, mast cells, endothelial cells, and smooth muscle cells (25–32). The diversity of productive and receptor-expressing cells is linked to the functional diversity of IL-18.

Like IL-1R, IL-18R contains a Toll/IL-1 receptor (TIR) domain in the intracellular region that is shared with TLRs, and signaling into the cell is mediated by myeloid differentiation factor 88 (MyD88) (33–35). MyD88 is a well-known adaptor molecule for TLRs and IL-1R. The activation of IL-18R results in the recruitment of MyD88 to the TIR and anchors IL-1 receptor-associated kinase (IRAK) (36). Phosphorylated IRAK dissociates from the complex and binds to tumor necrosis factor receptor-associated factor 6 (TRAF6), which in turn phosphorylates nuclear factor kappa B (NF-κB)-induced kinase (NIK) (37). This results in the activation of I kappa B (IκB) kinase (IKK). The phosphorylation of IκB by IKK leads to the ubiquitination and degradation of IκB (38). NF-κB is then able to migrate into the nucleus and initiate the transcription of target genes such as IFN-γ (39).

Although the major signaling pathway of IL-18 is NF-κB signaling, it has been reported that stimulation by IL-18 strongly promotes the tyrosine phosphorylation of STAT3 and the mitogen-activated protein kinases (MAPKs) p44erk-1 and p42erk-2 in human NK cell lines (40). In murine T cells, IL-18 induced the activation of the lymphocyte-specific tyrosine protein kinase p56lck and p42 MAPK (41).

## IL-18 Binding Protein

As with IL-1, the activity of IL-18 is regulated by the endogenous inhibitor IL-18 binding protein (IL-18BP). IL-18BPa, the major splice variant of IL-18BP, is present in excess concentrations compared to IL-18 in the serum of healthy individuals and it binds with high affinity to IL-18 to neutralize its activity (42–44). IL-18BP inhibits the binding of IL-18 to the IL-18 receptor and inhibits the production of IFN-γ (Figure 1). IFN-γ has been reported to mediate the gene expression of IL-18BPa in non-leukocytic cells (45). IL-18 activity is regulated by a negative feedback mechanism mediated by IL-18BPa induced by IFN-γ. It is thus likely that IL-18BPa functions as a “shut off” signal to stop the excessive inflammatory response by IL-18 (44). The expression of IL-18BP is regulated mainly at the transcriptional level, and signal transducer and activator of transcription 1 (STAT1) and CCAAT/enhancer binding protein β (C/EBPβ) have been reported to be important transcription factors in the regulation of IL-18BP gene promoter activity (46, 47).

The activity of IL-18 is balanced by the presence of IL-18BP. Serum IL-18BP levels are significantly elevated in sepsis and other inflammatory diseases (42–44, 48, 49). Patients with granulomatosis with polyangiitis (i.e., Wegener's granulomatosis) and those with systemic lupus erythematosus showed elevated serum levels of IL-18 as well as IL-18BP, but the levels of IL-18BP were not sufficient to neutralize IL-18, and the levels of free IL-18 were higher than those of healthy subjects (42, 48).

Exogenous IL-18BP may be useful as a novel therapeutic agent for diseases involving IL-18 (42–44, 48, 49). A phase II clinical trial was conducted in patients with adult-onset Still's disease (50); the administration of tadekinig, a recombinant IL-18BP, was observed to reduce the patients' serum C-reactive protein and ferritin levels and improve their clinical symptoms. A Phase III clinical trial of tadekinig is currently underway in patients suffering from pediatric monogenic auto-inflammatory diseases and harboring deleterious mutations of NLRC4 and XIAP (NCT03512314).

## Physiological Functions of IL-18

IL-18 was originally discovered as a factor that induces IFN-γ from Th1 cells. The most important role of IL-18 in the immune system is the induction of the production of IFN-γ by Th1 cells. IL-18 acts synergistically with IL-12 to induce a potent Th1 response (51–53), and IL-18 plays an important role in the host's defense mechanism against infections caused by pathogens such as bacteria, viruses, fungi, and protozoa (13). In concert with IL-12, IL-18 also induces the production of IFN-γ by NK cells, B cells, and macrophages (51, 54, 55). Although IL-18 induces IFN-γ production as a Th1 cytokine by co-stimulation with IL-12, IL-18 acts alone as a Th2 cytokine (13). Basophils and mast cells derived from bone marrow cells cultured with IL-3 for 10 days expressed IL-18Rα (56). IL-3 is involved in the differentiation of mouse bone marrow cells into basophils and mast cells (57). Basophils produced both of the Th2 cytokines IL-4 and IL-13 in response to stimulation with IL-3 + IL-18 (56). Although mast cells did not produce IL-4, they produced IL-13 in response to stimulation with IL-3 + IL-18 (56). The administration of IL-18 together with IL-12 inhibits both the production of IgE and the productions of IL-4 and IL-13 by basophils and mast cells in an IFN-γ-dependent manner (58). On the other hand, the administration of IL-18 alone has been reported to induce IgE production by B cells (59). IL-4 and IL-13 are involved in the production of IgE and the differentiation and proliferation of eosinophils and are important in the formation of allergic pathologies such as bronchial asthma and atopic dermatitis (60). These findings suggest that IL-18 may be involved in allergic inflammation.

In addition, IL-18 has been reported to up-regulate Fas ligand (FasL) expression in NK cells and induce apoptosis in Fas-positive target cells (61, 62). In NK cells, IL-18 also enhances perforin-mediated cytotoxic activity (32). The activation of NK cells suggested that IL-18 may be associated with tumor immune responses (13, 63). It has been reported that treatment with IL-18 in combination with the B7-1 costimulatory molecule resulted in the regression of melanoma with increased NK cell infiltration at the tumor tissue (64). In addition to the above-described activities, IL-18 induces the production of granulocyte/macrophage colony-stimulating factor (GM-CSF) and the expression of adhesion molecules. In co-culture with osteoblasts and hematopoietic cells, IL-18 inhibited the formation of osteoclastlike cells *via* the production of GM-CSF (65). IL-18 enhanced the expression of intercellular adhesion molecule-1 (ICAM-1) in human myelomonocytic cell lines (66), and ICAM-1 and vascular cell adhesion molecule-1 (VCAM-1) in endothelial cells and rheumatoid arthritis synovial fibroblasts (67). As described above, IL-18 has various pro-inflammatory effects besides the induction of IFN-γ production, and IL-18 may be associated with various pathologies such as infections, allergic diseases, and tumor immunity.

## IL-18 and Inflammatory Kidney Disease

Inflammation underlies the pathogenesis of many renal diseases, including acute kidney injury (AKI) and chronic kidney disease (CKD), and the role of IL-18 in inflammation has been reported in many experimental animal models (summarized in Table 1). In clinical practice, IL-18 is expected to be useful in the diagnosis of diseases and the estimation of disease severity and prognosis.

**Table 1:** IL-18 in renal disease models.

**Disease Model**	**Intervention**	**Outcome**	**References**
**AKI Model**			
IRI	IL-18 BP	Protective	(68)
IRI	IL-18-deficient	Protective	(69)
LPS	IL-18Rα-deficient	Protective	(70)
Cisplatin	Anti-IL-18 antibodies	Not protective	(71)
Cisplatin	Overexpression of IL-18BP	Not protective	(71)
Cisplatin	IL-18-deficient	Protective	(72)
Cisplatin	IL-18Rα-deficient	Detrimental	(73)
**CKD Model**			
Anti-GBM GN	IL-18-deficient	Protective	(74)
Immune complex GN	IL-18Rα-deficient	Protective	(75)
LN (MRL/lpr)	IL-18	Detrimental	(76)
LN (MRL/lpr)	IL-18Rα-deficient	Protective	(77)
LN (MRL/lpr)	IL-18-deficient	Protective	(78)
LN (MRL/lpr)	Anti-IL-18 autoantibodies (IL-18 vaccination)	Protective	(79)
UUO	Overexpression of IL-18BP	Protective	(80)
UUO	IL-18Rα-deficient	Protective	(81)

*IL, interleukin; IRI, ischemia-reperfusion injury; BP, binding protein; LPS, lipopolysaccharide; GBM, glomerular basement membrane; GN, glomerulonephritis; LN, lupus nephritis; UUO, unilateral ureteral obstruction*.

### IL-18 as a Biomarker for AKI

Urinary IL-18 has been reported to be increased in patients with acute tubular necrosis after kidney transplantation (82, 83). The urinary level of IL-18 is expected to be an early diagnostic marker of acute kidney injury (AKI), and many clinical trials have been conducted. A meta-analysis summarizing reports after cardiac surgery showed that the sensitivity and specificity values of urinary IL-18 as a biomarker for the diagnosis of AKI were 0.58 and 0.75, respectively (84). The area under the receiver operating characteristic curve (AUROC) of urinary IL-18 levels predictive of AKI was 0.70 (84). In another meta-analysis, the AUROC was similar at 0.77 (85). Urinary IL-18 is a biomarker of AKI with moderate diagnostic value. Although it does not reliably predict the development of AKI, urinary IL-18 has been reported to be useful to predict clinical outcomes including mortality and dialysis in a heterogeneous intensive care unit (ICU) population (86).

It has been reported that urinary IL-18 levels in patients with AKI after cardiopulmonary bypass increased over the first 4–6 h, peaked in 12 h, and remained elevated up to 48 h after surgery (87, 88). This elevation of urinary IL-18 in AKI is slower than that observed in urinary neutrophil gelatinase-associated lipocalin (87, 88). The urinary IL-18 level in patients with acute tubular necrosis has been shown to be significantly elevated compared to patients with urinary tract infections, pre-renal acute renal failure, chronic kidney disease, and nephrotic syndrome (82). However, because urinary IL-18 is also elevated in septic patients (86, 89), caution should be exercised when using the urinary IL-18 level for the diagnosis of AKI.

### Ischemic Renal Disease

Ischemia-reperfusion injury (IRI) in the kidney is used as a model of AKI. An AKI caused as a result of IRI involves both innate and acquired immune responses (90). In an IRI mouse model, the plasma and renal IL-18 levels were shown to be significantly increased after IRI stress (68, 69). Compared to wild-type mice, IL-18-deficient mice were protected from IRI and showed better renal function, less tubular damage, less neutrophil and macrophage infiltration, and less expression of downstream inflammatory mediators of IL-18 (69). In a mouse model of IRI, treatment with IL-18BP, an IL-18 inhibitor, showed a renal-protective effect (69). Treatment with IL-18 BP has also been shown to reduce the levels of profibrotic molecules in the kidneys of mice after IRI and to inhibit the progression of IRI-induced renal fibrosis (68). Because caspase-1 activates IL-18, caspase-1-deficient mice are also protected against ischemic acute renal failure (15). Although IL-18 is produced by various types of cells, it has been reported that cells of bone marrow origin play a more important role than intrinsic kidney cells in the renal damage caused by IRI (69). Blocking IL-18 signaling may be protective against IRI-induced AKI.

### LPS-Induced AKI

We reported the role of IL-18 in LPS-induced AKI in IL-18Rα-deficient mice (70). In CD4+ T cells derived from splenocytes, the mRNA expressions of IL-18 and IL-18Rα were significantly increased after LPS injection. The IL-18Rα-deficient mice showed lower blood urea nitrogen (BUN) levels, a higher survival rate, and reduced levels of pro-inflammatory cytokines such as IL-18 and IFN-γ compared to wild-type mice. Glomerular CD4+ T cells and interstitial macrophage infiltration were reduced in the kidneys of the IL-18Rα-deficient mice. IL-18R-mediated signaling pathways may plays critical roles in these cells in the pathogenesis of LPS-induced AKI.

### Cisplatin-Induced AKI

*In vitro*, cisplatin induces the apoptosis or necrosis of renal tubules (91). Cisplatin administration increases serum and renal levels of IL-18 (71). However, methods to inhibit IL-18 using IL-18 antiserum or transgenic mice that overproduce IL-18BP did not protect against cisplatin-induced AKI (71). On the other hand, IL-18-deficient mice have been reported to be protected from AKI, and the exogenous supplementation of recombinant IL-18 prior to cisplatin administration caused AKI (72). In our study using IL-18Rα-deficient mice, the inhibition of IL-18 signaling did not result in a favorable effect. We observed that compared to wild-type mice, the IL-18Rα-deficient mice had worse renal function and downregulated expressions of suppressor of cytokine signaling (SOCS) 1 and SOCS3 in the spleen and kidney (73). The inhibition of cytokine signaling by the members of the SOCS family constitutes a major negative feedback mechanism to prevent runaway inflammation. SOCS1 reduces the impact of cytokines by inhibiting JAK kinases and several other mechanisms (92). Although the mechanism is not clear, we speculate that IL-18Rα may induce an anti-inflammatory response by affecting the expressions of the cytokine signaling inhibitors SOCS1 and SOCS3 in addition to the inflammatory response. In summary, the inhibition of IL-18 may not be sufficient for the prevention of cisplatin-induced AKI. The effect of IL-18 on cisplatin-induced AKI appears to vary between mouse models, and further research is needed.

### Glomerulonephritis

Neutrophils play an important role in the pathogenesis of antineutrophil cytoplasmic antibody (ANCA)-associated vasculitis (AAV). Neutrophils require priming for subsequent ANCA-induced activation, and IL-18 is thought to be important for neutrophil priming in AAV, as is tumor necrosis factor-alpha (TNF-α) (93, 94). *In vitro*, IL-18 can prime neutrophils, and it enhances superoxide production by cells after ANCA binding (93, 94). AAV patients have higher serum IL-18 concentrations compared to healthy controls (95). In renal biopsies from AAV patients, IL-18-positive cells were found in podocytes in the glomerulus and in myofibroblasts, distal tubular epithelial cells, and infiltrating macrophages in the interstitium (94).

In patients with IgA nephropathy, serum IL-18 levels have been reported to correlate significantly with urinary protein excretion, serum creatinine, and the estimated glomerular filtration rate (eGFR) (96). In addition, patients with high IL-18 levels at baseline were shown to have worse renal function during the follow-up period (96). The serum IL-18 level may predict the reduction of renal function in patients with IgA nephropathy. In renal biopsies of IgA nephropathy patients, the expression levels of IL-18 were positively correlated with both the infiltration of inflammatory cells into the interstitium and the extent of proteinuria (74).

A role of IL-18 has been reported in several experimental animal models of glomerulonephritis. In a model of anti-glomerular basement membrane nephritis in mice, IL-18-deficient mice had reduced leukocyte infiltration in the glomeruli and interstitium (97). Based on our findings obtained with a bovine serum albumin glomerulonephritis mouse model, we reported that IL-18Rα-deficient mice showed a significant reduction of proteinuria, renal pathological findings including glomerular IgG and C3 deposits, and leukocyte infiltrates compared to control mice (75). Thus, in experiments with several animal models, the suppression of IL-18 signaling has been shown to be protective against glomerulonephritis.

### Lupus Nephritis

Lupus nephritis (LN) is a frequent and severe organ lesion associated with systemic lupus erythematosus (SLE) (98). IL-18 has been implicated in the pathogenesis of SLE, based on studies in mice and humans. MRL/lpr mice, which develop spontaneous lupus-like autoimmune disease, had higher levels of serum IL-18 compared to controls (76), and the mice treated with IL-18 developed accelerated proteinuria, glomerulonephritis and vasculitis (76). We reported that IL-18Rα-deficient MRL/lpr mice survived longer than IL-18α-intact MRL/lpr mice, and we observed significant reductions in glomerular IgG deposition, proteinuria, and serum anti-DNA antibodies in the IL-18Rα-deficient MRL/lpr mice (77). Similarly, some other groups have reported improved survival and proteinuria in IL-18-deficient and IL-18-vaccinated MRL/lpr mice (78, 79).

High serum IL-18 levels have been reported in patients with SLE (99, 100). Patients with active renal disease also have higher serum levels of IL-18 than those without renal activity (99, 100). It was reported that in the serum of LN patients, not only IL-18 but also IL-18BP, which neutralizes the activity of IL-18, are significantly increased, but the IL-18/IL-18BP ratio is also increased (101). This imbalance between IL-18 and IL-18BP may be involved in the pathogenesis of LN (101).

Serum IL-18 levels correlate with the disease activity of SLE (99, 100, 102) and are also associated with the severity of LN (100, 103, 104). Several studies indicated that the IL-18 value in the serum or glomeruli of patients with class IV LN was increased compared to the LN class III and V patients. Thus, IL-18 may be useful for the identification of LN in SLE patients and for estimating the severity of LN.

### Diabetic Nephropathy

Inflammatory cytokines play an important role in the development and progression of diabetic nephropathy (105). In clinical studies, elevated plasma and urinary IL-18 levels were associated with diabetic nephropathy, and IL-18 was observed to be a predictive marker for the development of diabetic nephropathy in diabetic patients and to be associated with the progression of renal dysfunction (106–108). Serum and urinary IL-18 levels correlate with the degree of urinary albumin excretion (107, 108). Elevated serum and urinary IL-18 levels in diabetic patients may be a risk factor for the development of diabetic nephropathy. In kidney tissue of diabetic nephropathy patients, IL-18 is overexpressed in tubular epithelial cells, which may occur *via* the activation of the MAPK pathways induced by transforming growth factor-beta (TGF-β) (109). Treatment that blocks IL-18 signaling may be a new approach in the treatment of diabetic nephropathy.

### Obstructive Nephropathy

Renal interstitial fibrosis is a common and important lesion in the process of various progressive renal diseases that progress to renal atrophy. Unilateral ureteral obstruction (UUO) is an important model for studying the mechanisms of renal fibrosis and evaluating the potential therapeutic approaches (110). In UUO model mice, it was reported that serum IL-18 levels were elevated and the renal IL-18 and IL-18R expressions were enhanced after a UUO operation (16, 17, 80, 81). We reported that compared to wild-type mice, IL-18Rα-deficient mice had reduced tubular cell apoptosis and suppressed renal interstitial fibrosis after UUO (81). Similarly, transgenic mice with neutralized IL-18 activity also show reduced fibrosis (80).

In general, TGF-β is a mediator that plays a central role in renal fibrosis (111). Interestingly, in our previous study, there was no significant difference in the expression of renal TGF-β between IL-18Rα-deficient and wild-type mice (81). IL-18 may be involved in renal interstitial fibrosis by a mechanism that is independent of TGF-β (80, 81). *In vitro*, FasL expression in human proximal tubular cells has been reported to be enhanced by IL-18 exposure, and IL-18 may stimulate proapoptotic signaling through a FasL-dependent mechanism and affect obstructive nephropathy (112). In addition, TLR4 signaling may affect IL-18-mediated profibrotic effects (113, 114). Experiments using these mouse models suggested that (i) IL-18 signaling plays an important role in renal interstitial fibrosis during renal obstruction, and (ii) the inhibition of IL-18 acts protectively against fibrosis.

### COVID-19

COVID-19, caused by severe acute respiratory syndrome coronavirus 2 (SARS-CoV-2) and first detected in the city of Wuhan in China's Hubei Province, has become a global pandemic. In response to SARS-CoV-2 infection, the human body produces pro-inflammatory cytokines such as IL-1β, IL-6, IL-7, IL-8, TNF-α, granulocyte-colony stimulating factor (G-CSF), interferon gamma-induced protein 10 (IP-10), monocyte chemotactic protein (MCP)-1, and MCP-3 (115–117). COVID-19 produces an excessive inflammation “cytokine storm” that leads to acute respiratory distress syndrome (ARDS) and the failure of multiple organs including kidney.

The incidence of AKI in COVID-19 patients is higher than that in non-COVID-19 patients, and AKI associated with COVID-19 has been shown to be independently associated with an almost 4-fold higher odds of death than AKI associated with other acute illnesses (118). However, in general, critically ill patients with ARDS and AKI have many complications that can induce acute tubulointerstitial injury, and the causal relationship between coronavirus infection and AKI remains unclear. Factors that may contribute to the development of AKI in COVID-19 patients include direct viral infections, the cytokine storm, drug treatments, hemodynamic instability, and advancing hypercoagulable state. In a renal histological analysis of samples from autopsies of 26 patients who died of COVID-19-induced respiratory failure, clinical signs of renal injury, including increased serum creatinine and/or new-onset proteinuria, were observed in only nine of the 26 patients (34.6%), while mild to severe acute tubular injury was observed in all 26 patients (119). Three patients had pigmented tubular casts, three had segmental glomerular fibrin thrombi and two had focal segmental glomerulosclerosis. In seven patients, electron microscopy showed coronavirus-like particles in the tubular epithelium and podocytes. The SARS-CoV-2 virus uses angiotensin-converting enzyme 2 (ACE2) as a receptor for host cell entry. In the kidney, ACE2 is predominantly expressed in proximal tubules and is also present in podocytes and endothelial and smooth muscle cells of vessels (120). The finding that SARS-CoV-2 infects these cells (119) may indicate that the virus causes renal injury directly. However, it has been reported that multivesicular bodies (MVBs) mimicking SARS-CoV-2 are found in podocytes of COVID-19-negative patients, and it has not yet been established whether SARS-CoV-2 truly causes direct kidney injury (121).

Many cytokines are involved in the pathogenesis of COVID-19, and IL-18 may also be relevant (122, 123). Serum IL-18 levels have been shown to correlate with serum IL-6 levels, with inflammatory markers such as C-reactive protein and ferritin, and with markers of organ injury such as creatinine, liver enzymes, and troponin (122). It has also been reported that serum IL-18 levels on admission are higher in COVID-19 patients requiring mechanical ventilation and lethal cases (123). IL-18 may be related to the severity of COVID-19. The appropriate control of pro-inflammatory cytokines, including IL-18, may be a therapeutic option for managing the complications caused by the cytokine storm in COVID-19. There are currently no clinical trials examining IL-18 signaling. On the other hand, the effect of the humanized anti-IL-6 receptor antibody tocilizumab on COVID-19 has been reported. Several open-label trials and non-randomized case series reported positive effects of tocilizumab on COVID-19 (124); however, phase III clinical trials did not show efficacy of tocilizumab for preventing intubation or death in moderately ill hospitalized patients with COVID-19 (125). Although the regulation of IL-18 signaling may be a potential therapeutic target for COVID-19, the suppression of IL-18 signaling alone may not be sufficient to control the disease, as many cytokines are involved in the severity of COVID-19.

## Conclusion

IL-18 belongs to the IL-1 superfamily and drives the same MyD88/NF-κB signaling pathway as IL-1β. IL-18 is a pro-inflammatory cytokine that induces IFN-γ production and has a variety of other functions, including the enhancement of NK cell activity and up-regulation of FasL expression. IL-18 appears to regulate inflammation at multiple checkpoints. Pre-clinical and clinical studies have obtained interesting results in many circumstances in which IL-18 is associated with an increased inflammatory infiltrate and more severe kidney lesions. These results suggest that IL-18 may play an important role in the pathology of inflammatory kidney diseases, and they raise expectations that IL-18 may be a potential therapeutic target. However, there is a lack of clinical studies targeting IL-18 in inflammatory renal disease. In addition, the role and signaling of IL-18 in inflammatory kidney disease are not fully understood. It remains unknown whether IL-18 is clearly implicated in disease pathogeneses. In experimental animal models, IL-18-deficiency, anti-IL-18 antibodies, IL-18R-deficiency, and IL-18BP all regulate IL-18 signaling, and in many cases their effects are protective for the kidneys. Some conflicting results suggest that their respective signaling pathways, effects on cytokines, etc. may not be identical. Studies that will further elucidate IL-18 signaling are important for understanding the pathogenesis of inflammatory kidney disease and for therapeutic applications.

## Author Contributions

YH drafted the manuscript. YN edited the manuscript. Both authors contributed to the manuscript's revision and have read and approved the submitted version.

## Conflict of Interest

The authors declare that the research was conducted in the absence of any commercial or financial relationships that could be construed as a potential conflict of interest.
